# Speech acoustics, prefrontal activation, and their coupling during affective prosody production in dementia of the Alzheimer’s type

**DOI:** 10.3389/fnagi.2026.1937665

**Published:** 2026-10-30

**Authors:** Chorong Oh, In-Sop Kim

**Affiliations:** 1 Department of Communication Sciences and Disorders, University of Kentucky, Lexington, KY, United States; 2 Division of Communication Sciences and Disorders, Ohio University, Athens, OH, United States; 3 School of Clinical and Developmental Sciences, Northern Illinois University, DeKalb, IL, United States

**Keywords:** affective prosody, dementia, emotion, fNIRS, multimodal sensing, prefrontal cortex, speech acoustics, speech biomarkers

## Abstract

**Introduction:**

Affective prosody, the expression of emotion through speech, is often disrupted in dementia of the Alzheimer’s type (DAT), yet its acoustic and neural bases remain poorly understood. Extending our earlier single-group investigation, we compared individuals with DAT and cognitively healthy older adults across speech acoustics, prefrontal activation, and their coupling.

**Methods:**

Ten individuals with DAT and ten cognitively healthy older adults of similar mean age produced spontaneous happy, sad, and neutral speech while prefrontal cortical activity was recorded using functional near-infrared spectroscopy (fNIRS). Acoustic features were extracted and analyzed alongside cortical activation and speech–brain activation coupling.

**Results:**

The groups differed reliably in the formant domain, while intensity, fundamental frequency, and formant structure varied by affective condition. Overall prefrontal activation did not differ between groups, but group-by-emotion interactions revealed opposite response directions: healthy adults generally showed increased activation during affective speech, whereas individuals with DAT showed decreased activation. Seven of eight contrasts survived correction for multiple comparisons. The pattern was replicated in total hemoglobin and remained robust to the removal of individual participants. Speech–brain activation coupling also differed between groups, with large effect sizes (Cohen’s *q* = 0.64–0.66), although these exploratory effects did not survive correction for multiple comparisons.

**Discussion:**

DAT was characterized not by uniform reduction but by reorganization of the relationship between affective speech and its cortical support. Joint analysis of acoustic and neural signals may provide a more comprehensive understanding of affective communication in dementia, while the exploratory coupling findings require confirmation in a larger sample.

## 1 Introduction

Affective prosody, or the modulation of pitch, loudness, tempo, and voice quality that conveys a speaker’s emotional state, is a foundation of human communication. It allows listeners to grasp intent and feeling beyond the literal content of an utterance and is therefore central to social connection (Banse and Scherer, 1996; Scherer, 2003). A large body of work has shown that emotions are encoded in speech through systematic acoustic patterns spanning frequency, intensity, timing, and voice quality (Juslin and Laukka, 2003; Holland and Kensinger, 2010). Unlike linguistic prosody that marks syntactic and pragmatic structure, affective prosody communicates how a speaker feels, and its disruption can carry tangible interpersonal costs.

The neural systems supporting affective prosody have been studied primarily through perception. Converging evidence implicates a distributed, right-weighted network in which the superior temporal cortex differentiates emotional from neutral speech, and the bilateral inferior frontal gyrus (IFG) supports the categorization and evaluation of vocal emotion (Frühholz and Grandjean, 2012; Grandjean et al., 2005; Wildgruber et al., 2004; Zhang et al., 2018). Lesion and imaging studies likewise emphasize right-hemisphere and inferior-frontal contributions to prosodic processing, though the degree of lateralization varies with task and stimulus (Mitchell and Ross, 2008; Pell and Leonard, 2003; Ross and Monnot, 2008). Far less is known about affective prosody production. The few neuroimaging studies that have examined emotional speech generation also point to the IFG and to interacting limbic and basal-ganglia circuits, with IFG activity scaling with the ability to convey the intended emotion (Mitchell et al., 2016; Patel et al., 2018; Pichon and Kell, 2013). Because the production and perception of emotion do not rely on identical mechanisms, and because everyday communication depends on speaking as much as listening, research into the neural basis of emotional speech production is an urgent need.

In Alzheimer’s disease and related disorders, affective prosody is of particular interest because it sits at the intersection of cognitive, communicative, and social decline. The existing literature, however, has concentrated on perception, with mixed conclusions: some studies report impaired comprehension of affective prosody (Taler et al., 2008; Testa et al., 2001), whereas across related dementia syndromes others find emotional processing relatively preserved (Kumfor and Piguet, 2012; Rohrer et al., 2012). Expressive affective prosody has received significantly less attention, and the work that exists suggests reduced emotional modulation of speech in dementia (Horley et al., 2010). In parallel, a rapidly growing literature treats acoustic features of spontaneous speech as scalable, non-invasive biomarkers of cognitive decline (Amini et al., 2023, 2024; Oh et al., 2023), underscoring the diagnostic value of characterizing how speech changes in dementia.

### 1.1 Relationship to our prior work

The present study is a direct extension of our earlier report (Oh et al., 2025), in which we combined acoustic analysis with functional near-infrared spectroscopy (fNIRS), a quiet, portable modality well suited to studying speech in clinical populations (Bendall et al., 2016), to characterize affective prosody production and its cortical correlates in individuals with DAT. That study was a single-group, within-DAT investigation: it established that affective prosody production could be captured concurrently at the acoustic and cortical levels in this population, but it had no comparison group and therefore could not address how the acoustic or neural signature of emotional production in DAT differs from that of normal aging. The DAT participants’acoustic and fNIRS data reported here are the same data reported in Oh et al. (2025). The cognitively healthy comparison group is reported here for the first time, and none of the analyses presented in this manuscript appear in the earlier report.

The addition of a healthy comparison group is not an incremental extension but a change in the questions that can be asked. Three questions that were unanswerable in a single-group design become tractable. First, although behavioral studies have compared emotional speech production in DAT with that of healthy controls (Horley et al., 2010; Testa et al., 2001), the few studies pairing acoustic with neural measurement have examined DAT alone, leaving it unknown how the neural signature of emotional production differs from that of normal aging, a question that requires a control group by definition. Second, even studies with acoustic measurement have focused on frequency, intensity, and timing, leaving voice-quality and spectral features, established correlates of vocal emotion, largely unexamined (Gobl, 2003; Horley et al., 2010; Testa et al., 2001). Third, and most fundamentally, acoustic and neural measures have been treated as parallel but separate descriptions, so that whether the two are linked during emotional production, and whether that link is altered in dementia, has not been investigated.

### 1.2 Speech–brain activation coupling

Producing affective prosody is inherently a joint process: the acoustic output and the cortical activity that generates it are two aspects of a single act. We refer to the degree to which variation in acoustic features tracks variation in prefrontal activation during emotional speech as speech–brain activation coupling. Describing acoustics and activation separately asks whether each differs by group; examining their coupling asks a categorically different question: whether the relationship between what is expressed and how the brain supports expressing it is itself restructured in disease. To our knowledge, this relationship has not been quantified in either healthy aging or dementia.

### 1.3 The present study

Using spontaneous affective and non-affective speech (happy, sad, and neutral conditions) recorded with simultaneous prefrontal fNIRS, we compared cognitively healthy older adults with individuals with DAT across three analytic layers. We asked: (1) Does a comprehensive acoustic feature set distinguish the two groups and track affective condition? (2) Does prefrontal activation differentiate the groups and vary with affective condition? (3) Does speech–brain activation coupling diverge between groups? We hypothesized group differences in the acoustic expression of affect, altered prefrontal activation in DAT, and reduced or reorganized coupling in DAT relative to healthy aging.

Given the inferior-frontal and right-weighted emphasis of the prosody literature, analyses centered on bilateral inferior frontal and frontopolar regions. Layers 1 and 2 are treated as confirmatory. Because the sample is modest, Layer 3 (coupling) is framed as exploratory, and we report effect sizes together with a formal sensitivity analysis specifying the sample size a confirmatory test would require.

## 2 Materials and methods

### 2.1 Participants

All procedures were reviewed and approved by the Institutional Review Board at Northern Illinois University (HS23-0135) and conducted in accordance with applicable ethical and regulatory standards. The study enrolled two groups of older adults: cognitively healthy adults and individuals with dementia of the Alzheimer’s type (DAT).

Across both groups, participants were required to (1) be at least 65 years old and (2) be native speakers of English. For the cognitively healthy group, eligibility additionally required an education-adjusted score on the Saint Louis University Mental Status (SLUMS) examination (Tariq et al., 2006) of at least 25 for those without a high school diploma, or at least 27 for high school graduates. Healthy participants were excluded if any of the following applied: (1) current use of medication for a psychiatric or mental-health condition; (2) a self-reported active, disruptive psychotic symptom such as delusional thinking; (3) a diagnosed neurological disorder; (4) a prior brain injury; (5) hearing loss not adequately corrected by hearing aids; or (6) current smoking.

Participants in the dementia group were recruited through community partners and all had DAT diagnosed by a board-certified neurologist; their cognitive status was further characterized by a SLUMS score in the 4–20 range, consistent with mild-to-moderate impairment. For these participants, a caregiver confirmed the dementia diagnosis, provided information on current medications, and gave informed consent on the participant’s behalf.

Ten cognitively healthy older adults (6 female, 4 male) and ten individuals with DAT (5 female, 5 male) met these criteria and completed all study tasks. Demographic and screening characteristics are summarized in Table 1. The DAT group scored substantially lower than the healthy group on the SLUMS (*M* = 15.4, SD = 6.4 vs. *M* = 27.5, SD = 0.7; Welch’s *t*(9.2) = 5.94, *p* < 0.001). The groups were comparable in education and smoking history (Table 1); no participant in either group was a current smoker. Mean age was similar across groups (*M* = 71.5, SD = 6.3 for DAT vs. *M* = 69.3, SD = 2.6 for healthy; Welch’s *t*(11.9) = −1.02, *p* = 0.328, *d* = −0.46), but we do not claim the groups were age-equivalent: a nonsignificant test at this sample size cannot establish equivalence, and the DAT group spanned a wider range (65–87) than the healthy group (65–73). Age was therefore included as a mean-centered covariate in all models reported below, and its treatment is discussed in section “4.5 Age.”

**TABLE 1:** Demographic and screening characteristics by group.

Characteristic	Healthy (*n* = 10)	DAT (*n* = 10)
Sex, F/M	6/4	5/5
Age, M (SD)	69.3 (2.6)	71.5 (6.3)
Age range	65–73	65–87
Education, n		
Code 2: High School	3	3
Code 3: Some college	6	5
Code 4: Bachelor’s degree	0	1
Code 5: Graduate degree	1	1
Smoking history, *n* (former/never)	2/8	4/6
SLUMS, M (SD)	27.5 (0.7)	15.4 (6.4)
SLUMS range	27–29	4–20
PANAS-Positive, M (SD)	34.0 (3.8)	28.8 (11.2)
PANAS-Negative, M (SD)	13.0 (3.2)	15.6 (5.8)
Race	White	White
Ethnicity	Non-Hispanic	Non-Hispanic

SLUMS, Saint Louis University Mental Status examination; PANAS, Positive and Negative Affect Schedule, administered immediately before the speech tasks; DAT, dementia of the Alzheimer’s type. Group comparisons: age, Welch’s *t*(11.9) = −1.02, *p* = 0.328; SLUMS, *t*(9.2) = 5.94, *p* < 0.001; PANAS-Positive, *t*(11.1) = 1.39, *p* = 0.191; PANAS-Negative, *t*(13.9) = −1.24, *p* = 0.235; education, χ^2^ *p* = 0.78; smoking history, Fisher’s exact *p* = 0.63. Hearing adequacy (with correction if used), absence of psychiatric medication, absence of active psychotic symptoms, and non-smoking status were eligibility criteria for the healthy group and were verified at screening; for the DAT group, caregivers reported current medications at enrollment. Disease duration, formal language assessment, and standardized functional-severity staging were not collected (section “4.8 Limitations”).

The acoustic and fNIRS data from the DAT group were previously reported in Oh et al. (2025), a single-group exploratory study of affective prosody in DAT. The cognitively healthy participants are reported here for the first time, and all analyses presented in this manuscript: the between-group acoustic contrasts, the group × emotion interaction in prefrontal activation, and the speech–brain coupling analyses are novel to the present report.

### 2.2 Procedure

Immediately before the speech tasks, participants completed the Positive and Negative Affect Schedule (PANAS; Watson et al., 1988) to index pre-task mood. The groups did not differ on either subscale (PANAS-Positive: healthy *M* = 34.0, SD = 3.8; DAT *M* = 28.8, SD = 11.2; Welch’s *t*(11.1) = 1.39, *p* = 0.191; PANAS-Negative: healthy M = 13.0, SD = 3.2; DAT *M* = 15.6, SD = 5.8; *t*(13.9) = −1.24, *p* = 0.235), although the DAT group was more variable in positive affect. Each participant produced spontaneous speech under three elicitation conditions that varied in emotional content. In the Happy and Sad conditions, participants were asked to recall and describe a personally significant life experience associated with the target emotion. In the Neutral condition, they explained, step by step, how to make a peanut-butter-and-jelly sandwich, as though guiding someone who had never done so. Eliciting emotion through autobiographical recall was chosen because re-experiencing a remembered event reinstates the associated affective and physiological state, and these states in turn shape vocal output and prosodic structure (Holland and Kensinger, 2010; Murphy et al., 2021). The sandwich-description task served as an affectively neutral comparison: recounting an overlearned procedure relies on automatized routines supported by subcortical circuitry and tends to yield matter-of-fact speech with little emotional coloring (Lum et al., 2012; Squire and Dede, 2015; Ullman, 2004). Relative to scripted reading or sentence-repetition paradigms, this task set was intended to approximate the demands of ordinary conversation and thereby improve ecological validity. The order of the three tasks was randomized across participants, and a short rest was given between tasks. Speech was captured in a sound-attenuated room with a Sony PCM-M10 portable recorder held 10 cm from the mouth at a 45-degree angle.

### 2.3 Acoustic analysis

Audio was prepared for analysis through a two-step automated workflow. Each recording was first transcribed with OpenAI’s Whisper speech-recognition system (large-v3 model; Radford et al., 2023). The transcripts and their waveforms were then submitted to the Montreal Forced Aligner (MFA, v3.3.9; McAuliffe et al., 2017) with the US-English ARPA pronunciation dictionary and acoustic model, yielding word- and phoneme-level TextGrids. Before alignment, transcripts were checked and lightly corrected by hand.

Per-utterance acoustic measurements were obtained with a purpose-written Python routine that calls Praat (v6.4; Boersma and Weenink, 2024) through the Parselmouth library (Jadoul et al., 2018). Rather than relying on Praat’s default settings, we tuned the extraction parameters for aged voices: the pitch floor and (sex-specific) pitch ceiling, the harmonics-to-noise ratio estimation routine, the formant ceiling, pause-detection and silence thresholds (the latter fixed at −25 dB), the syllable-counting procedure, the analysis window for spectral-tilt measures, and the MFCC window and step size were all adjusted accordingly. Because intensity-peak methods of estimating tempo are unreliable when amplitude modulation is reduced, as is common in older speakers, speech rate was instead derived from the phoneme boundaries returned by MFA. The agreement between this automated pipeline and manual annotation was evaluated in a separate methodological study, with the manuscript submitted for publication.

In total, 61 features were computed for every utterance and consolidated, following the structure of the extended Geneva Minimalistic Acoustic Parameter Set (eGeMAPS; Eyben et al., 2016), into seven feature domains: fundamental frequency (F0 mean, SD, minimum, maximum); intensity (mean, SD, minimum, maximum); voice quality (five jitter indices, six shimmer indices, and mean and SD of harmonics-to-noise ratio); formants (F1–F4); spectral source/tilt (H1–H2, H1–A1, H1–A2, H1–A3, and the component amplitudes H1, H2, A1, A2, A3); temporal characteristics (mean pause duration, pauses per utterance, speech rate); and MFCC (mean and SD of MFCCs 1–12). Because the domains differ in dimensionality, each was represented two ways: as the mean of its z-standardized constituent features and as its first principal component and the two representations were compared as a robustness check. We note that the first principal component of the MFCC domain accounted for only 33% of that domain’s variance, indicating that any single-value summary of this 24-dimensional domain is a weak representation; this bears on the interpretation of MFCC-based coupling effects (section “4.4 Interpreting the MFCC coupling effect”).

### 2.4 fNIRS acquisition and preprocessing

Cortical hemodynamics were recorded while participants performed the speech tasks, using the acquisition protocol described in detail by Oh et al. (2025). Briefly, prefrontal activity was monitored with a 16-channel continuous-wave fNIRS system (fNIR 400, Biopac) operating at 730 and 850 nm, with the optode band positioned bilaterally across the forehead to cover prefrontal cortex (Figure 1). Data were acquired at 2 Hz through COBI Studio and fNIRSOFT. Recordings were screened visually to verify scalp–optode contact and to flag hair-related interference (raw values above 4,000 mV or below 400 mV), then low-pass filtered at 0.1 Hz; motion and saturation artifacts were removed with sliding-window motion-artifact rejection (lower and upper bounds of 3 and 50). A 30-s rest preceded each task to allow the hemodynamic signal to return toward baseline, consistent with standard fNIRS practice (Yang and Hong, 2021). Oxygenated hemoglobin (HbO) was the primary index, with deoxygenated (HbR) and total (HbT) hemoglobin retained for secondary analyses.

**FIGURE 1:**
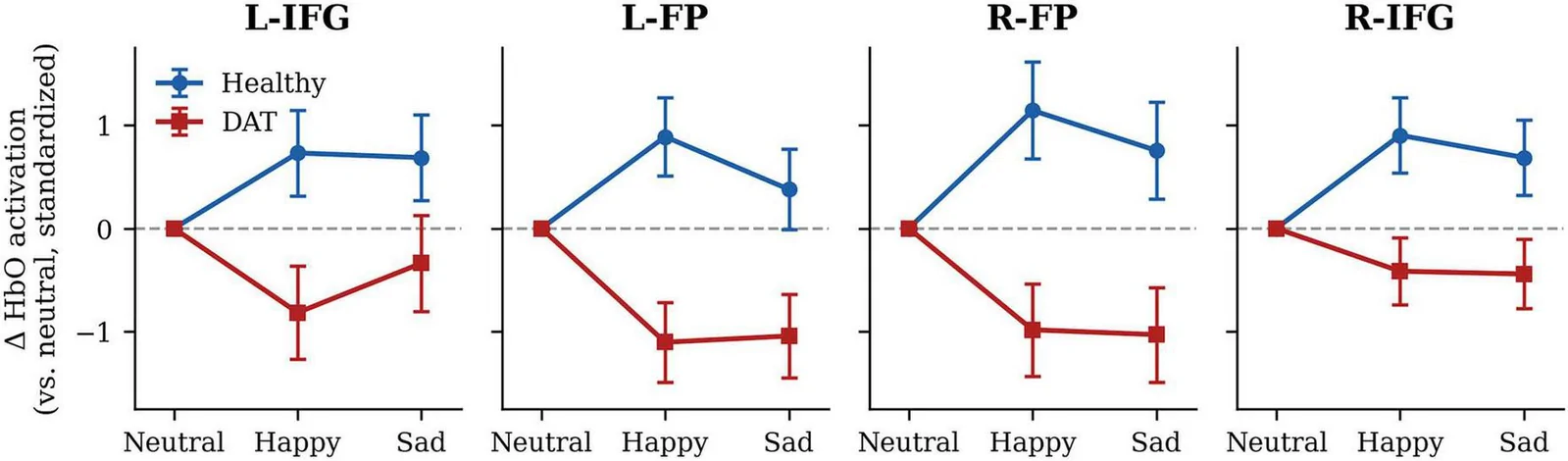
Optode locations. Sixteen-channel prefrontal montage, grouped into four regions of interest: left inferior frontal gyrus (L-IFG, channels 1–4), left frontopolar cortex (L-FP, channels 5–8), right frontopolar cortex (R-FP, channels 9–12), and right inferior frontal gyrus (R-IFG, channels 13–16).

The 16 channels were grouped into four anatomically motivated regions of interest (ROIs): left and right inferior frontal cortex (L-IFG, channels 1–4; R-IFG, channels 13–16) and left and right frontopolar cortex (L-FP, channels 5–8; R-FP, channels 9–12). This parcellation was specified *a priori* because the inferior frontal gyrus is repeatedly implicated in the production and evaluation of emotional prosody and in language production more broadly (Frühholz and Grandjean, 2012; Zhang et al., 2018). For each participant, condition, and chromophore, channel values were averaged over the task block and then within ROI, yielding one value per ROI per condition.

### 2.5 Missing data

Two sources of missing data affected the fNIRS dataset and are reported here in full. First, one affective condition (Sad) for one DAT participant was excluded because of a data-export anomaly, so that the DAT Sad cell reflects 9 rather than 10 participants; the fNIRS dataset therefore comprises 59 rather than 60 participant × condition cells. Second, optode dropout reduced usable cells in two ROIs: the right frontopolar ROI (R-FP) lost 7 of 59 cells (11.9%, leaving *n* = 52), and the left frontopolar ROI (L-FP) lost 2 cells (leaving *n* = 57); the two inferior-frontal ROIs had complete data (*n* = 59). Affected cells were excluded listwise from models involving the respective ROI. A breakdown of dropout by group and condition is given in Supplementary Table 1. Dropout was distributed across both groups (R-FP: 3 of 30 healthy cells and 4 of 29 DAT cells; L-FP: 2 of 30 healthy and 0 of 29 DAT) and across all three conditions, with no concentration in any group × condition cell. Because R-FP contributes to two of our principal findings [i.e., the largest group × emotion interaction (Table 2) and the temporal coupling effect (Table 3)], R-FP results should be interpreted with correspondingly greater caution. As reported in section “3.2 Prefrontal activation by group and emotion,” the same directional pattern is present in the two complete-data ROIs, indicating that the central finding does not depend on the R-FP data.

**TABLE 2:** Group × emotion interaction in prefrontal HbO, by ROI and emotion condition (age-covaried).

ROI	Emotion	Healthy β	DAT β	Interaction β	P (FDR)
L-FP	Happy	+0.89	−1.10	−2.00	0.004
	Sad	+0.38	−1.04	−1.42	0.022
R-FP^†^	Happy	+1.14	−0.98	−2.14	0.004
	Sad	+0.76	−1.03	−1.79	0.017
L-IFG	Happy	+0.73	−0.82	−1.55	0.022
	Sad	+0.69	−0.34	−1.01	0.112
R-IFG	Happy	+0.90	−0.42	−1.32	0.018
	Sad	+0.69	−0.44	−1.12	0.033

Within-group columns give standardized fixed-effect estimates for each affective condition relative to neutral; positive values indicate increased activation. The interaction column gives the group × emotion estimate (DAT relative to healthy); negative values indicate that the healthy increase is reversed in DAT. *p* (FDR), Benjamini–Hochberg-corrected across the eight contrasts. Age was included as a covariate.

† R-FP estimates are based on 52 of 59 cells owing to optode dropout; L-FP on 57 of 59 (section “2.5 Missing data”). IFG, inferior frontal gyrus; FP, frontopolar cortex.

**TABLE 3:** Speech–brain activation coupling: domain × ROI effects converging across both analytic approaches (exploratory).

Domain × ROI	r (healthy)	r (DAT)	Cohen’s q	P (Fisher)	P (mixed)	Power
Temporal × R-FP^†^	+0.37	−0.27	0.66	0.025	0.024	0.61
MFCC × L-FP	+0.14	−0.46	0.64	0.023	0.003	0.63

*r*, within-group Pearson correlation between the acoustic domain and ROI HbO, computed across participant × condition cells. *p* (Fisher), between-group Fisher *z*-test; this test treats cells as independent and its *p*-values are therefore optimistic (section “2.6.3 Layer 3: exploratory speech–brain coupling”). *p* (mixed), Group × Acoustic interaction from the age-covaried mixed-effects model that also includes an Emotion × Group term (section “2.6.3 Layer 3: exploratory speech–brain coupling”), which models within-participant dependency and is the primary analysis. Cohen’s *q*, difference between Fisher-transformed correlations; *q* ≥ 0.50 is conventionally large (Cohen, 1988). Power is the achieved power at the realized cell count, α = 0.05. Neither effect survived FDR correction across the 28 tests in this layer.

† R-FP estimates are based on 52 of 59 cells (section “2.5 Missing data”).

### 2.6 Statistical analysis

Analyses proceeded in three layers. All models were estimated as linear mixed-effects models with a by-participant random intercept, fitted by maximum likelihood so that nested models could be compared by likelihood-ratio tests; emotion condition (Neutral as reference) and group (Healthy as reference) were entered as fixed effects, and mean-centered age was included as a covariate throughout. Within each analytic layer, *p*-values were adjusted using the Benjamini–Hochberg false discovery rate (FDR) procedure.

#### 2.6.1 Layer 1: acoustic

Layer 1 tested whether each of the seven acoustic domains varied by group and emotion, modeled at the utterance level (566 utterances nested within participants): domain ∼ Group × Emotion + Age + (1 — Participant).

#### 2.6.2 Layer 2: prefrontal activation

Layer 2 tested whether prefrontal HbO varied by group and affective condition. Each ROI was modeled as HbO ∼ Group × Emotion + Age + (1 — Participant). The group × emotion interaction was evaluated in two complementary ways. First, a likelihood-ratio test compared the full interaction model against a reduced main-effects model within each ROI. Second, the emotion effect was re-estimated within each group separately to characterize its direction. The same interaction model was applied to HbR and HbT to assess robustness across chromophores.

#### 2.6.3 Layer 3: exploratory speech–brain coupling

Layer 3 tested whether the acoustic–neural relationship differed between groups. Because fNIRS was acquired in a block design, this layer is resolved at the participant × condition level rather than the utterance level; acoustic domain scores were averaged within each participant × condition cell before being related to the corresponding ROI value.

The primary analysis was a mixed-effects moderation model, HbO ∼ Acoustic domain × Group + Emotion × Group + Age + (1 — Participant), in which the Group × Acoustic interaction was the term of interest; the by-participant random intercept explicitly accounts for the dependency among the three cells contributed by each participant. The Emotion × Group term was included so that the task-evoked hemodynamic difference between groups established in Layer 2 is modeled explicitly, and the Group × Acoustic term therefore tests whether the acoustic–neural slope differs between groups over and above any group difference in the emotion effect itself. As a descriptive cross-check, within-group Pearson correlations between each acoustic domain and each ROI were computed and compared between groups using Fisher’s *r*-to-*z* transformation. We note explicitly that this correlational cross-check treats participant × condition cells as independent observations and therefore does not model within-participant dependency; its *p*-values are consequently optimistic, and it is reported only as a descriptive complement to the moderation model. Convergence of the two approaches on the same domain–ROI pairs, despite their different treatment of dependency, is the basis for identifying candidate effects.

#### 2.6.4 Sensitivity and robustness analyses

Two additional analyses were conducted. First, to quantify the power available for the coupling layer, we computed Cohen’s *q* (the difference between Fisher-transformed correlations; Cohen, 1988), the achieved power at the realized cell count, and the number of participants per group needed to detect the observed effects at 80% power, both at α = 0.05 and under a Bonferroni-equivalent worst-case adjustment for the 28 domain × ROI tests. Second, to assess whether findings depended on any individual participant, a natural concern at this sample size, we conducted leave-one-subject-out (LOSO) analyses in which each model was refitted 20 times, omitting one participant each time.

Analyses were conducted in Python 3.12 (statsmodels, SciPy, scikit-learn). Principal-component representations of the acoustic domains were used to confirm that conclusions were not contingent on the choice of domain summary.

## 3 Results

Results are presented in three layers: the speech signal (section “3.1 Acoustic differences by group and emotion”), prefrontal hemodynamics (section “3.2 Prefrontal activation by group and emotion”), and their coupling (section “3.3 Exploratory speech–brain activation coupling”), followed by sensitivity and robustness analyses (sections 3.4–3.5). All models included mean-centered age as a covariate, and *p*-values were FDR-corrected within each layer. Layers 1 and 2 are confirmatory; Layer 3 is exploratory and is labeled as such throughout.

### 3.1 Acoustic differences by group and emotion

At the utterance level (566 utterances from 20 speakers), the groups differed in the formant domain, which was the only domain to survive FDR correction (β = 0.73, *p*FDR = 0.005), with the DAT group producing higher formant domain scores than healthy speakers. Temporal (β = 0.28, *p*FDR = 0.056), spectral source/tilt (β = 0.31, *p*FDR = 0.128), and intensity (β = 0.38, *p*FDR = 0.128) showed group effects at the trend level; fundamental frequency, voice quality, and MFCC did not differ between groups (Table 4).

**TABLE 4:** Group main effects (DAT vs. healthy) for each acoustic domain, age-covaried.

Acoustic domain	β	P	P (FDR)	Group difference
Formants (F1–F4)	0.73	0.001	0.005	DAT > healthy *
Temporal (rate/pause)	0.28	0.016	0.056	Trend
Intensity	0.38	0.063	0.128	Trend
Spectral source/tilt	0.31	0.073	0.128	Trend
Voice quality (jitter/shimmer/HNR)	−0.20	0.268	0.375	ns
MFCC	−0.07	0.361	0.410	ns
Fundamental frequency (F0)	0.20	0.410	0.410	ns

Positive β indicates higher domain scores in DAT. Models are utterance-level mixed-effects models with a by-participant random intercept and mean-centered age as a covariate. *p* (FDR), Benjamini–Hochberg-corrected within the acoustic layer.

*pFDR < 0.05. ns, not significant.

Emotion modulated the acoustic signal robustly and did so most clearly in the sad condition. Relative to neutral, sad speech showed reliably lower fundamental frequency (β = −0.40, *p*FDR = 0.0001) and intensity (β = −0.43, *p*FDR < 0.0001), higher formant scores (β = 0.33, *p*FDR = 0.005), and altered spectral source measures (β = −0.15, *p*FDR = 0.028). The happy condition reliably altered intensity only (β = −0.20, *p*FDR = 0.038). This asymmetry (i.e., sad speech being more acoustically differentiated from neutral than happy speech) is consistent with prior reports in healthy older adults.

### 3.2 Prefrontal activation by group and emotion

Considered as a main effect, group did not modulate HbO activation in any ROI (all *p* > 0.24): overall activation magnitude did not separate individuals with DAT from healthy older adults. This null main effect, however, masked a systematic difference in how the two groups responded to affective condition. Age, entered as a covariate, was non-significant in every ROI (all *p* > 0.15).

A group × emotion interaction was tested in each ROI. The interaction survived FDR correction in seven of eight contrasts (Table 2), and likelihood-ratio tests confirmed that allowing the emotion effect to vary by group improved model fit in three of four ROIs (L-FP, χ^2^(2) = 10.92, *p* = 0.004; R-FP, χ^2^(2) = 10.54, *p* = 0.005; R-IFG, χ^2^(2) = 7.18, *p* = 0.028; L-IFG at trend, χ^2^(2) = 5.82, *p* = 0.055).

The form of the interaction was consistent across all four regions and is the central neural finding of the study. In healthy older adults, affective conditions were associated with increased prefrontal activation relative to neutral (Happy: R-FP β = +1.14; R-IFG β = +0.90; L-FP β = +0.89; L-IFG β = +0.73). In the DAT group, the same conditions were associated with decreased activation (Happy: L-FP β = −1.10; R-FP β = −0.98; L-IFG β = −0.82; R-IFG β = −0.42). Because the two groups moved in opposite directions (Figure 2), their activation magnitudes averaged out, accounting for the absent group main effect. Rather than a uniform reduction in activation, DAT was characterized by a reversal in the direction of the prefrontal response to affective speech. Critically, this pattern is present in the two complete-data ROIs (L-IFG and R-IFG, both *n* = 59) as well as in the two frontopolar ROIs affected by optode dropout, so it does not depend on the incomplete R-FP data.

**FIGURE 2:**
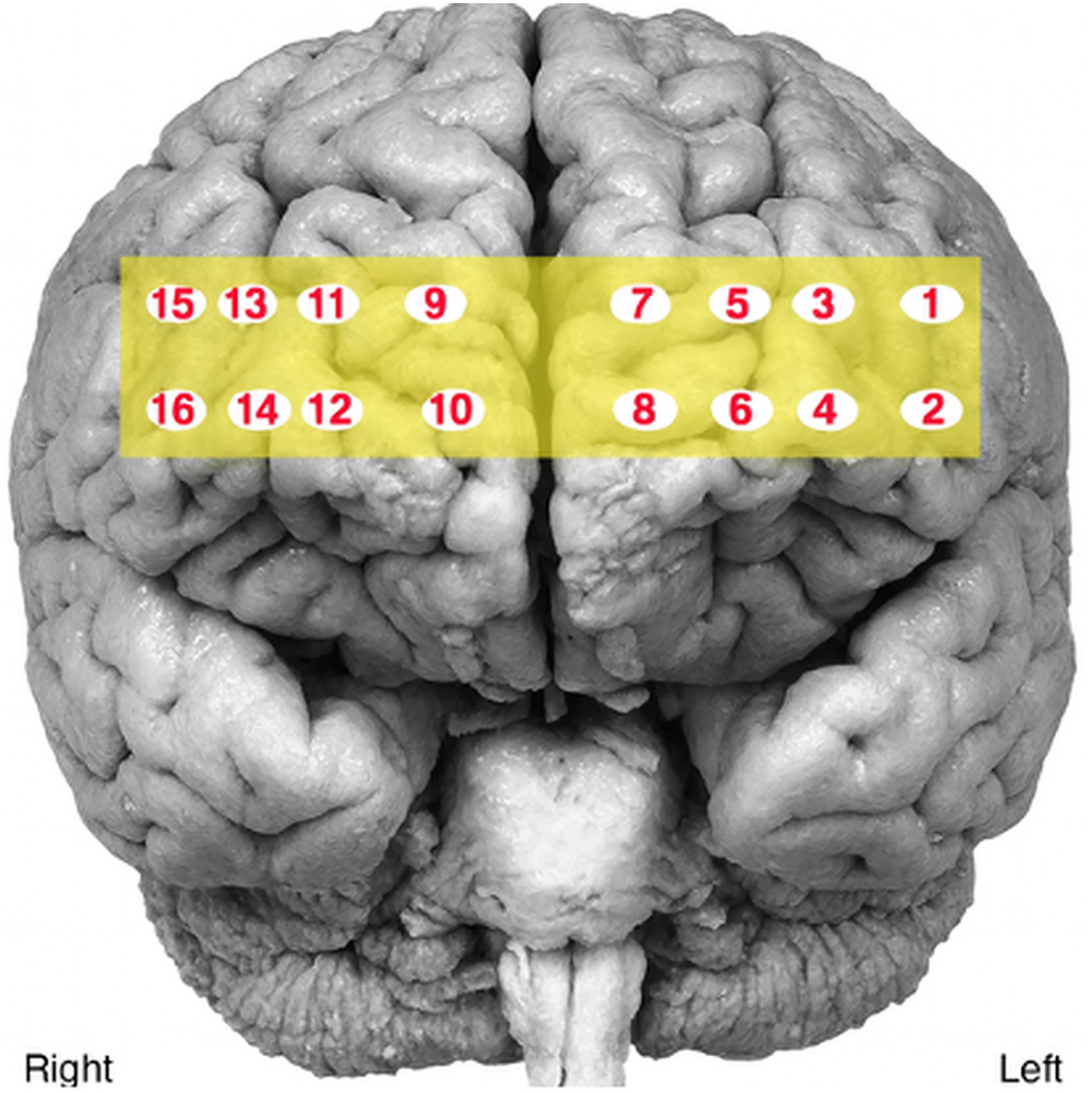
Within-group prefrontal HbO response to affective conditions relative to neutral, by region of interest. Points are model-estimated marginal effects (age-covaried); error bars are standard errors. Healthy older adults show increased activation during affective speech, whereas individuals with DAT show decreased activation, yielding a group × emotion interaction that survived FDR correction in seven of eight contrasts. R-FP estimates are based on 52 of 59 participant × condition cells and L-FP on 57 of 59, owing to optode dropout. L-/R-IFG, left/right inferior frontal gyrus; L-/R-FP, left/right frontopolar cortex.

Because HbO was the primary chromophore, we examined whether the same interaction was present in HbR and HbT. In HbT the pattern paralleled HbO: healthy older adults showed increased frontopolar activation during affective speech while individuals with DAT showed decreased activation, and the group × emotion interaction survived FDR correction in both frontopolar regions (L-FP, β = −3.03, *p*FDR = 0.022; R-FP, β = −3.37, *p*FDR = 0.022), with likelihood-ratio tests confirming improved fit (L-FP, χ^2^(2) = 7.37, *p* = 0.025; R-FP, χ^2^(2) = 9.48, *p* = 0.009). In HbR, no interaction survived correction and likelihood-ratio tests were non-significant across regions. The weaker HbR effects are expected given the lower signal-to-noise ratio of that signal in fNIRS (Strangman et al., 2002), and the convergence of HbO and HbT indicates that the directional reversal is carried by the oxygenated component. Full statistics are given in Table 5.

**TABLE 5:** Group × emotion (happy vs. neutral) interaction for HbR and HbT, by ROI (age-covaried).

ROI	HbR β	HbR p	HbR p(FDR)	HbT β	HbT p	HbT p(FDR)
L-FP	−1.05	0.242	0.431	−3.03	0.011	0.022*
R-FP^†^	−1.23	0.128	0.431	−3.37	0.006	0.022*
L-IFG	+0.32	0.787	0.787	−1.23	0.449	0.449
R-IFG	+0.49	0.323	0.431	−0.83	0.318	0.425

β, Group × Happy interaction estimate (DAT relative to healthy); negative values indicate that the healthy increase is reversed in DAT. *p*(FDR), Benjamini–Hochberg-corrected within each chromophore.

*pFDR < 0.05. Age was included as a covariate.

† R-FP estimates are based on 52 of 59 cells (section “2.5 Missing data”). HbR, deoxygenated hemoglobin; HbT, total hemoglobin.

### 3.3 Exploratory speech–brain activation coupling

This layer is exploratory: no effect survived FDR correction across the 28 domain × ROI tests (minimum *p*FDR = 0.070), and the results below are reported as directional estimates to guide confirmatory work, not as established effects. The mixed-effects moderation model (which accounts for within-participant dependency and, as specified in section “2.6.3 Layer 3: exploratory speech–brain coupling,” allows the emotion effect to differ between groups) and the correlational cross-check (which does not) converged on two domain–ROI pairs (Table 3). In both cases the direction was the same: healthy speakers showed a positive acoustic–neural association, whereas the DAT group showed a negative association. The alignment between speech output and prefrontal engagement that characterized healthy aging was thus reduced, and in these cells reversed, in dementia. Two further cells reached *p* < 0.05 in the moderation model: MFCC × R-FP (*p* = 0.016), which did not reach significance in the cross-check (*p* = 0.056) and is not treated as convergent, and MFCC × L-IFG (*p* = 0.037; cross-check *p* = 0.044), which met the convergence criterion but was less stable under leave-one-subject-out refitting (16 of 20 folds) and is noted here rather than added to Table 3. Principal-component representations of the acoustic domains yielded the same pattern, indicating that the result was not an artifact of the domain-summary method.

### 3.4 Sensitivity analysis for the coupling layer

Because the coupling effects did not survive correction, we quantified what the present design could and could not detect. Both convergent effects were large by conventional standards (Cohen’s *q* = 0.64 and 0.66; *q* ≥ 0.50 is designated large; Cohen, 1988), yet the realized design afforded only 61%–63% power to detect them at α = 0.05 uncorrected. The smallest between-group difference the present design could detect at 80% power corresponds to *q* = 0.76, larger than either observed effect. The coupling results are therefore best characterized not as weak or unreliable effects, but as large effects observed in an underpowered design.

Table 6 translates this into a concrete target. Detecting the temporal effect (*q* = 0.66) at 80% power would require approximately 13 participants per group at α = 0.05, or approximately 25 per group under a conservative worst-case correction for the 28 tests in this layer. These are attainable sample sizes, and they constitute a specific, falsifiable prediction that a replication study can test.

**TABLE 6:** Robustness and sensitivity of the reported effects.

Effect	Cohen’s q	Power	*n*/group (α = 0.05)	*n*/group (corr.)	
(A) Sensitivity: participants per group required for 80% power					
Temporal × R-FP	0.66	0.61	13	25	
MFCC × L-FP	0.64	0.63	14	27	
**Effect**	**β**	***P***	**LOSO β range**	**LOSO max *p***	**Folds *p* < 0.05**
(B) Leave-one-subject-out robustness (20 folds)					
Layer 2: group × emotion (happy)					
L-FP	−2.00	0.0005	−2.20 to −1.65	0.0017	20/20
R-FP^†^	−2.14	0.0010	−2.47 to −1.84	0.0041	20/20
R-IFG	−1.32	0.0090	−1.55 to −1.07	0.0315	20/20
L-IFG	−1.55	0.0136	−1.85 to −1.19	0.0512	19/20
Layer 3: coupling (exploratory)					
Temporal × R-FP^†^	−1.29	0.024	–	0.1217	18/20
MFCC × L-FP	−2.32	0.003	–	0.0331	20/20

(A) Power and required *n* computed for the between-group comparison of Fisher-transformed correlations; “current” power assumes the realized cell counts, and required *n* per group assumes three emotion conditions per participant. “Corrected” applies a Bonferroni-equivalent worst-case adjustment for the 28 domain × ROI tests (α = 0.05/28), and is therefore conservative relative to the Benjamini–Hochberg procedure actually used. (B) Each model was refitted 20 times, omitting one participant per fold. β is the group × emotion (happy) estimate for Layer 2 and the Group × Acoustic interaction estimate for Layer 3. LOSO β range is omitted for Layer 3, where the coefficient scale differs across domains.

† R-FP estimates are based on 52 of 59 cells owing to optode dropout (section “2.5 Missing data”).

### 3.5 Leave-one-subject-out robustness

A natural concern at this sample size is that findings might be driven by one or two individuals. To test this directly, every model was refitted 20 times, omitting one participant each time (Table 6B). The group × emotion interaction in prefrontal activation was significant in 20 of 20 folds in three ROIs (L-FP, R-FP, R-IFG) and in 19 of 20 folds in L-IFG; the interaction estimate varied only modestly across folds (e.g., L-FP range −2.20 to −1.65; R-FP range −2.47 to −1.84). The central neural finding therefore does not depend on any individual participant. The exploratory coupling effects were likewise stable (Temporal × R-FP significant in 19 of 20 folds; MFCC × L-FP in 18 of 20), although they remained below the FDR threshold in the full sample.

## 4 Discussion

This study compared affective prosody production in dementia of the Alzheimer’s type and cognitively healthy older adults across three analytic layers: speech acoustics, prefrontal activation, and the coupling between them. The recurring theme was not uniform loss but reorganization: the acoustic signature of affect differed between groups, the direction of the prefrontal response to affective speech reversed in DAT, and the acoustic–neural coupling diverged between groups. We interpret each layer in turn before arguing that their convergence is what makes the pattern interpretable.

### 4.1 Acoustic findings

The formant domain was the only acoustic domain to distinguish the groups after correction, with temporal, intensity, and spectral measures showing trends in the same direction. The locus of this difference is not where earlier studies looked. Whereas prior acoustic work emphasized fundamental frequency and timing (Oh et al., 2025; Testa et al., 2001) and found the clearest DAT deficits in pitch modulation (Horley et al., 2010), our strongest group effect was in formant structure, and F0 did not differ between groups at all. Two clarifications are needed. First, formants are not the primary acoustic carriers of vocal affect in the broader literature (Juslin and Laukka, 2003; Scherer, 2003), and in our data emotion-related modulation was largest in F0 and intensity, although the formant and spectral-source domains were also modulated by emotion (Section 3.1). The formant domain was thus sensitive to both emotion and group, but it was the only domain on which the groups differed after correction (Table 4). We therefore interpret the formant effect not as an index of affective prosody *per se*, but as an acoustic dimension on which the groups differed while producing affective speech. Formants are nonetheless not irrelevant to emotion: affective arousal and valence alter vocal-tract configuration, raising F1 and F2, respectively, and the vowel space varies with emotion dimensions (Goudbeek et al., 2008, 2009; Goudbeek and Scherer, 2010), so a group difference in this channel during affective speech is meaningful even though its mechanism is not established here. Second, a combined z-score across F1–F4 has no directional articulatory interpretation, and we do not claim that the higher DAT score reflects imprecise articulation. Vowel-space compression of the kind documented in motor-speech disorders (Sapir et al., 2010; Rusz et al., 2013) is one hypothesis, but distinguishing an articulatory change from differences in the vowels produced or in speaker vocal-tract characteristics requires a controlled-vowel task, which we identify as a priority for follow-up (section “4.8 Limitations”). What the present data do show is that, when a comprehensive feature set is examined, the clearest group separation is not in the pitch and timing measures emphasized by earlier work but in formant structure, consistent with the broader move toward rich acoustic profiling as a sensitive marker of cognitive change (Amini et al., 2023, 2024).

The emotion effects were, by contrast, both strong and asymmetric. Sad speech was reliably differentiated from neutral across four domains (i.e., F0, intensity, formants, and spectral source), whereas happy speech differed reliably in intensity alone. That sad prosody is more acoustically uniform, and more clearly marked, than happy prosody is consistent with what we and others have found in healthy older adults, and suggests that the two affective states are not equivalently encoded in the speech signal.

### 4.2 The prefrontal reversal

The neural findings are, on their face, paradoxical and demand interpretation. Overall prefrontal activation did not differ between groups, yet the response to affective condition was reversed: healthy older adults showed increased prefrontal activation during affective relative to neutral speech, whereas individuals with DAT showed decreased activation. This pattern held across all four ROIs, survived correction in seven of eight contrasts, was replicated in total hemoglobin, and as the LOSO analysis establishes, was robust to the removal of any individual participant. The hypothesis that prefrontal activation would differ in DAT was thus supported, but in a more specific form than anticipated: as a reversal of direction rather than a change in overall magnitude.

Three complementary frameworks help situate this pattern. First, models of compensatory scaffolding hold that healthy aging is accompanied by increased prefrontal recruitment that supports performance in the face of declining neural resources (Cabeza, 2002; Park and Reuter-Lorenz, 2009; Reuter-Lorenz and Cappell, 2008). On this view, the prefrontal increase we observed in healthy older adults during affective speech is itself a compensatory signature, and its reversal in DAT may reflect a failure of that scaffolding when pathology exceeds the brain’s compensatory capacity. Second, the framework of neural dedifferentiation proposes that aging and disease reduce the selectivity of neural responses, so that activation patterns become less distinct across conditions (Park et al., 2004; Reuter-Lorenz et al., 2000). The collapse, and inversion, of the normal affective-versus-neutral distinction in DAT is consistent with a loss of response specificity in prefrontal cortex. Third, these accounts are not mutually exclusive: a compensatory response that is selectively recruited in health may be precisely what dedifferentiation erodes in disease. Our cross-sectional design cannot adjudicate among these mechanisms, and the reversal should be read as a robust phenomenon in search of a mechanism, and we do not adjudicate among these accounts. We offer these frameworks as possibilities, not as demonstrated mechanisms. In particular, greater prefrontal activation is not by itself evidence of successful compensation; that reading would require showing that the additional recruitment supported performance, and we did not relate activation to a behavioral outcome in this study. Where we use the term reorganization in what follows, it denotes a change in the observed relationship between speech output and prefrontal hemodynamics, not a claim about the neural process that produced it.

The opposite prefrontal HbO responses observed between healthy adults and individuals with DAT during affective prosody production should not necessarily be interpreted as evidence of cortical reorganization. These differences may reflect variations in cognitive effort, attentional and memory demands, or neurovascular regulation associated with DAT. Specifically, reduced HbO responses in individuals with DAT may indicate diminished neural recruitment, altered task engagement, or impaired neurovascular coupling rather than functional reorganization. Although the group-by-emotion interaction was statistically robust and showed a similar pattern in total hemoglobin, the underlying mechanisms remain uncertain. Therefore, these findings are more cautiously interpreted as altered task-related prefrontal hemodynamic responses during affective prosody production in DAT.

Furthermore, the emotional and neutral speech tasks differed in their memory retrieval demands. Emotional tasks required recalling autobiographical experiences, whereas the neutral task involved describing a familiar procedure. Consequently, differences in prefrontal hemodynamic responses may partly reflect autobiographical memory retrieval difficulties and increased cognitive demands in individuals with DAT rather than affective prosody production alone. Future studies using emotionally varied tasks with comparable memory demands may help distinguish emotion-related neural responses from those associated with memory retrieval.

Finally, distinguishing functional cerebral activity from speech-related motion (e.g., jaw movement) and systemic physiological noise (e.g., breathing, scalp blood flow) is a critical consideration in fNIRS speech paradigms. Although signal preprocessing included 0.1 Hz low-pass filtering and sliding-window motion-artifact rejection, residual systemic noise remains a potential confound. The convergence of findings across oxygenated (HbO) and total (HbT) hemoglobin supports a true hemodynamic origin rather than optode decoupling artifacts. Nevertheless, because continuous-wave fNIRS without short-separation channels cannot fully isolate cerebral from superficial scalp blood flow, these findings are cautiously interpreted as altered task-related prefrontal hemodynamic responses during affective prosody production in DAT.

### 4.3 Speech–brain coupling: a large effect in an underpowered design

Where healthy speakers showed positive coupling between acoustic features and prefrontal activation, individuals with DAT showed negative coupling. Reduced coupling resonates with the long-standing characterization of Alzheimer’s disease as a disconnection syndrome, in which the integrity of communication between brain regions, and between brain and behavior, is progressively degraded (Delbeuck et al., 2003). While the disconnection literature has largely concerned cortico-cortical functional connectivity, the present results extend the logic to the link between a behavioral output, the speech signal, and its cortical correlate. That the coupling reversal mirrors the activation reversal is mutually reinforcing: both point to a breakdown in the orderly relationship between affective speech and prefrontal engagement.

Reviewer attention to the direction of these associations is warranted, and we clarify what it means for each domain. The temporal domain score is the mean of z-standardized pause duration, pauses per utterance, and speech rate; because the two pause measures dominate the composite (correlations with the composite of 0.73 and 0.79 at the participant × condition level, versus 0.10 for speech rate), a higher temporal score indicates more and longer pausing, not faster speech. The positive coupling in healthy older adults (*r* = +0.37) therefore means that more pausing accompanied greater right frontopolar activation, a pattern consistent with pausing during spontaneous affective speech reflecting planning and retrieval supported by prefrontal cortex. In DAT the sign reversed (*r* = −0.27): more pausing accompanied less frontopolar activation, so that the pauses which in healthy speakers co-occurred with prefrontal engagement did not do so in DAT. The MFCC domain summarizes spectral-envelope shape and has no direct physiological reading (section “4.4 Interpreting the MFCC coupling effect”), so we interpret its coupling only as a second instance of the same directional divergence. A negative association is not in itself weaker or worse than a positive one; what we take to be informative is that the sign differs between groups for the same acoustic measure, which is what a changed relationship between speech output and its prefrontal support would produce. Whether this bears on participants’ability to express emotion is not something the present data can establish: we did not obtain independent perceptual ratings of the emotional expressiveness of the speech samples, and we identify such ratings as a necessary component of the confirmatory study.

No coupling effect survived FDR correction, and we do not claim that coupling reorganization in DAT is established. What the sensitivity analysis (section “3.4 Sensitivity analysis for the coupling layer”) shows is that this null is a property of the design rather than of the effects: the convergent effects were large by conventional standards, but the realized design was underpowered to detect them, and the minimum effect detectable at 80% power exceeded both. A study of roughly 25 participants per group would be powered to test the temporal effect under correction. We therefore report the coupling layer as a quantified, falsifiable hypothesis with an attached sample-size requirement.

### 4.4 Interpreting the MFCC coupling effect

One of the two convergent coupling effects involved the MFCC domain, and it warrants separate comment. Statistically it was comparable to the temporal effect in magnitude (Cohen’s *q* = 0.64 vs. 0.66) and slightly stronger in the moderation model. We nonetheless interpret it with greater caution, for two reasons.

First, the first principal component of the MFCC domain accounted for only 33% of that domain’s variance, meaning that any single-value summary of this 24-dimensional feature set is a weak representation of it; the coupling estimate is correspondingly imprecise about which aspect of the MFCC space carries the effect. Second, and more fundamentally, MFCCs are a cepstral encoding of spectral envelope shape with no direct physiological or articulatory interpretation. A coupling between MFCC variation and prefrontal hemodynamics is therefore difficult to situate mechanistically: we can describe it, but we cannot say what it means. By contrast, the temporal domain, speech rate and pause structure, has a clear interpretive route, since the timing of spontaneous speech depends on executive and planning processes supported by frontopolar cortex, making a Temporal × R-FP coupling both measurable and mechanistically legible. We therefore foreground the temporal effect in theoretical discussion and report the MFCC effect for completeness and directional consistency, without building theoretical claims upon it. Should confirmatory work replicate the MFCC coupling, identifying which MFCC dimensions carry the effect would be a necessary next step.

### 4.5 Age

As noted in section “2.1 Participants,” mean age was similar across groups but we do not treat them as age-equivalent: the test is underpowered for that purpose, and the DAT group’s age range (65–87) was substantially wider than the healthy group’s (65–73). Age was therefore entered as a mean-centered covariate in every model, where it was non-significant in all four ROIs (all *p* > 0.15) and left the group × emotion interaction unchanged.

Two further considerations bear on interpretation, neither of which we regard as decisive. First, the DAT group was on average slightly older (by 2.2 years). Compensatory-scaffolding accounts predict increased prefrontal recruitment with advancing age (Cabeza, 2002; Reuter-Lorenz and Cappell, 2008); if age alone were driving the result, the older group would be expected to show greater, not lesser, prefrontal activation during affective speech. The observed decrease in DAT is thus not the direction an age-based account would predict, which reduces, though does not eliminate, the plausibility of that account. Second, the group main effect on prefrontal activation was null in every ROI (all *p* > 0.24): an age-driven difference in overall prefrontal recruitment would be expected to appear there and did not. We nonetheless regard the age distribution as a limitation, and age matching, with a narrower and overlapping range in both groups, is a design requirement for the confirmatory study specified in Table 6.

### 4.6 Why the layers must be read together

The central contribution of this study is what becomes visible only when the three layers are considered together. Each, alone, would mislead. Acoustic analysis alone documents that DAT speech differs but is silent on whether the brain’s involvement is altered. Activation analysis alone, had it stopped at the group main effect, would have concluded that prefrontal engagement is preserved in DAT, missing the reversal entirely, because the opposing group responses cancel when averaged. Coupling analysis alone, underpowered here, would have offered only a suggestive signal. It is the convergence of altered acoustics, reversed activation, and divergent coupling that licenses a coherent interpretation: affective prosody production in DAT is marked by a reorganization of the relationship between speech and its cortical support, not by a simple attenuation of either. The cancellation in the activation main effect is a concrete cautionary case: a unimodal or main-effects-only analysis would have reported a null where a structured group difference in fact exists (Figure 3). We note that this argument is available to us only because a comparison group was added; it could not have been made from the single-group data reported previously (Oh et al., 2025).

**FIGURE 3:**
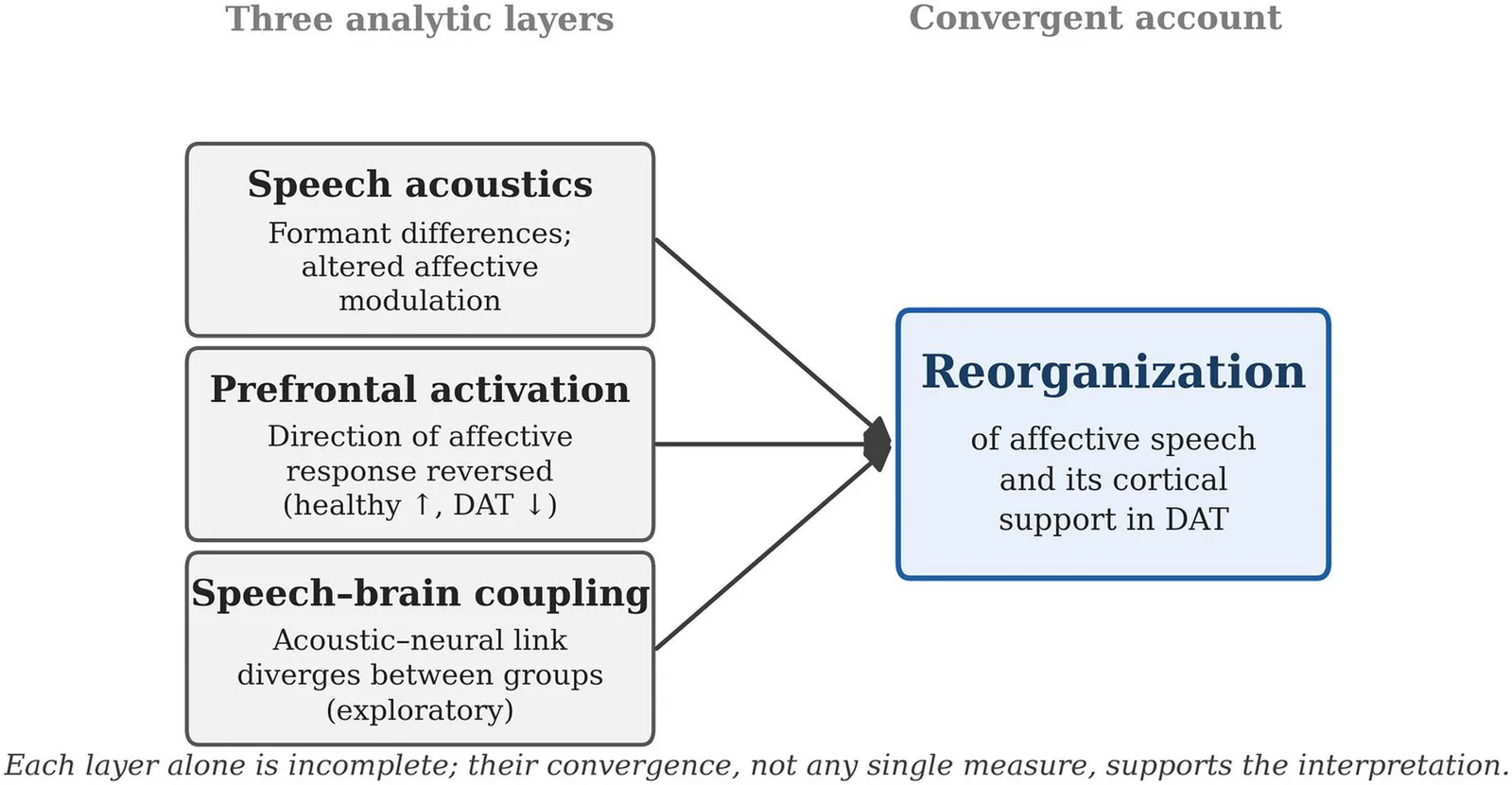
Schematic of the three analytic layers and their convergence. Differences in speech acoustics, a reversal in the direction of the prefrontal affective response, and divergent speech–brain coupling each point toward a reorganization of affective speech production in DAT. Layers 1 and 2 are confirmatory; the coupling layer (Layer 3) is exploratory and did not survive correction for multiple comparisons.

### 4.7 Implications

These findings carry clinical and methodological implications. Clinically, reduced or reversed modulation of affective prosody may contribute to the communication and social-connection difficulties experienced by individuals with DAT and their caregivers, and the formant-based features identified here are candidate markers for non-invasive monitoring.

The prefrontal reversal, however, complicates a premise on which speech-based markers of cognitive decline implicitly rest. Acoustic features are attractive precisely because they are scalable and non-invasive, and their diagnostic use presumes that a change in the speech signal indexes a change in the neural processes that generate it. Our data indicate that this mapping is not fixed. Overall prefrontal engagement was statistically indistinguishable between groups, yet the two groups arrived at that equivalence through opposite responses to affective content; had we measured activation alone, we would have concluded that prefrontal recruitment is preserved in DAT. By extension, comparable acoustic output need not imply comparable neural production: the same surface signal may be generated by a reorganized process. This does not undermine the value of speech markers, but it does bound their interpretation. A marker that tracks output is not thereby a marker of mechanism, and the two can dissociate in precisely the population the marker is meant to serve. Establishing what a speech feature indexes neurally, rather than assuming it, is therefore part of validating it.

Methodologically, the results argue for pairing acoustic with neural measurement in studies of communication in dementia, and for treating the coupling between modalities as a target in its own right rather than as two parallel descriptions. Speech–brain coupling is one tractable way to give a sensor-derived signal a neural referent, and the sample-size estimates in Table 6 provide a concrete design specification for the confirmatory work that would be required to establish it.

### 4.8 Limitations

Several limitations qualify these conclusions; the most consequential are addressed in dedicated sections above (sections “3.4 Sensitivity analysis for the coupling layer,” 4.3–4.5). Six further limitations warrant statement.

First, the sample was modest (10 per group). Layers 1 and 2 were adequately powered for the effects observed: the acoustic contrasts rest on 566 utterances, and the activation reversal was large, replicated across chromophores, consistent across all four ROIs, and robust to the removal of any participant; but the coupling layer was not, as quantified above. Second, optode dropout affected the frontopolar ROIs (R-FP, 7 of 59 cells; L-FP, 2 of 59), and R-FP carries both the largest interaction estimate and the temporal coupling effect; that the same directional pattern appears in the two complete-data ROIs mitigates but does not eliminate this concern. Third, the design was cross-sectional, so the reorganization observed here cannot be tied to disease progression, nor can the compensation and dedifferentiation accounts be distinguished. Fourth, fNIRS coverage was confined to prefrontal cortex, whereas affective prosody production engages broader cortical and subcortical networks, including basal-ganglia and temporal contributions (Pichon and Kell, 2013); extending spatial coverage is an important next step. Fifth, the formant group difference rests on spontaneous speech in which vowel content was not controlled, and formant values depend on vocal-tract length and therefore on speaker characteristics. Sex was closely balanced across groups (healthy 6F/4M, DAT 5F/5M), which mitigates but does not remove this concern, and the effect should be replicated with a controlled-elicitation task before an articulatory interpretation is adopted. Sixth, disease duration, formal language assessment, and standardized functional-severity staging were not collected in this protocol; dementia status was established by neurologist diagnosis and severity was characterized by SLUMS, and standardized staging is a priority for the confirmatory study.

### 4.9 Conclusion

Affective prosody production in DAT appears to be characterized not by uniform loss but by a reorganization evident across speech acoustics, prefrontal activation, and their coupling. Each modality offered a distinct and partial view; only jointly did they yield a coherent account, and in at least one instance a unimodal analysis would have been misleading. The prefrontal reversal is, on the present evidence, a robust phenomenon: large, replicated across chromophores, consistent across regions, and independent of any individual participant. The coupling reorganization is, on the present evidence, a large but underpowered signal with a specified sample-size requirement for confirmation. Distinguishing clearly between these two epistemic statuses is, we believe, more useful to the field than treating them alike. Together they establish the feasibility and the interpretive payoff of a multimodal, coupling-based approach to affective communication in dementia, and they hand a subsequent study a concrete target.

## Data Availability

The raw data supporting the conclusions of this article will be made available by the authors, without undue reservation.
