# Supplementary material for: Speech acoustics, prefrontal activation, and their coupling during affective prosody production in dementia of the Alzheimer’s type
Source: Front Aging Neurosci. 2026 Oct 30;18:1937665. doi: 10.3389/fnagi.2026.1937665 (PMC13759779; doi:10.3389/fnagi.2026.1937665)
Supplement: Supplementary file 1 [file Table_1.docx]

**Supplementary Material**

Speech acoustics, prefrontal activation, and their coupling during affective prosody production in dementia of the Alzheimer’s type

Oh C, Kim IS, et al.

**SUPPLEMENTARY TABLE S1 |** Optode dropout (missing participant × condition cells) by ROI, group, and condition.

| **ROI** | **Group** | **Neutral** | **Happy** | **Sad** | **Usable / attempted** |
| --- | --- | --- | --- | --- | --- |
| L-IFG | Healthy | 0 | 0 | 0 | 30 / 30 |
|  | DAT | 0 | 0 | 0 | 29 / 29 |
| L-FP | Healthy | 1 | 0 | 1 | 28 / 30 |
|  | DAT | 0 | 0 | 0 | 29 / 29 |
| R-FP | Healthy | 1 | 1 | 1 | 27 / 30 |
|  | DAT | 2 | 1 | 1 | 25 / 29 |
| R-IFG | Healthy | 0 | 0 | 0 | 30 / 30 |
|  | DAT | 0 | 0 | 0 | 29 / 29 |

Cells give the number of participant × condition observations excluded for that ROI owing to insufficient scalp–optode contact. Attempted cells are 30 per group (10 participants × 3 conditions) except DAT, where one Sad cell was excluded for an unrelated data-export anomaly (Section 2.5), leaving 29. DAT, dementia of the Alzheimer's type; IFG, inferior frontal gyrus; FP, frontopolar cortex.
